# Supplementary material for: Behavioral thermoregulation by reptile embryos promotes hatching success and synchronization
Source: Commun Biol. 2023 Aug 15;6:848. doi: 10.1038/s42003-023-05229-8 (PMC10427690; doi:10.1038/s42003-023-05229-8)
Supplement: Supplementary file 2 — Supplmentary tables [file 42003_2023_5229_MOESM2_ESM.pdf]

Table S1. Effects of capsazepine on embryonic development and hatchling traits in the Chinese soft-shelled turtle, *Pelodiscus sinensis*

Variable	Control	Capsazepine	Statistical analysis
Incubation period (days)	44.42 ± 0.02 (n = 90)	44.39 ± 0.02 (n = 92)	$F_{1,167}$ = 0.875; p = 0.351
Coefficient of variation	0.0012 ± 0.0007 (n = 15)	0.0011± 0.0007 (n = 15)	$F_{1,28}$ = 0.008; p = 0.929
Variation range (days)	0.13 ± 0.07 (n = 15)	0.12 ± 0.08 (n = 15)	$F_{1,28}$ = 0.009; p = 0.923
Hatching success (%)	85.7 ± 3.40 (n = 15)	87.6 ± 4.20 (n = 15)	$F_{1,28}$ = 0.126; p = 0.726
Carapace length (mm)	27.01 ± 0.14 (n = 90)	26.91 ± 0.13 (n = 92)	$F_{1,182}$ = 0.467; p = 0.495
Carapace width (mm)	24.31 ± 0.16 (n = 90)	24.13 ± 0.14 (n = 92)	$F_{1,182}$ = 1.463; p = 0.288
Body mass (g)	3.37 ± 0.05 (n = 90)	3.36 ± 0.04 (n = 92)	$F_{1,182}$ = 1.055; p = 0.402
Righting time (s)	0.52 ± 0.01 (n = 90)	0.52 ± 0.01 (n = 92)	$F_{1,166}$ = 0.315; p = 0.575

We evenly divided eggs from the same clutch to control and capsazepine groups. The effect of capsazepine on hatching success and righting time were analyzed by generalized linear mixed models with the clutch number as the random factor. Effects of capsazepine on traits of hatchlings were analyzed by linear mixed models with the clutch number as the random factor and initial egg mass as the covariate. Data are presented with means ± SE.

Table S2. Effects of behavioral thermoregulation on hatchling traits in *Pelodiscus sinensis*

Variable	Thermoregulation	Thermoregulation-inhibited	Statistical analysis
Body mass (g)	3.32 ± 0.03 (n = 215)	3.30 ± 0.02 (n = 203)	$F_{1,370} = 0.251$; $p = 0.617$
Righting time (s)	0.54 ± 0.01 (n = 214)	0.54 ± 0.01 (n = 202)	$F_{1,374} = 0.711$; $p = 0.400$

Effects of behavioral thermoregulation on traits of hatchlings were analyzed by linear mixed models with the clutch number as the random factor and initial egg mass as the covariate. Data are presented with means ± SE.

Table S3. The difference in thermal environments among open, filtered, and fully-shaded habitats

Mean air and soil surface temperatures above the nest in different habitats.

	Open habitat	Filtered habitat	Shaded habitat
Air	28.66 ± 0.23°C (n = 768)	28.32 ± 0.21°C (n = 768)	27.49 ± 0.18°C (n = 768)
Soil surface	31.05 ± 0.38°C (n = 768)	29.08 ± 0.21°C (n = 768)	26.74 ± 0.16°C (n = 768)

The Kruskal-Wallis test was used to evaluate the air and surface temperatures of semi-natural experimental sites, and the temperature heterogeneity of different nest locations, data show as mean ± SE. During embryonic development, the air temperatures (Kruskal-Wallis chi-squared = 9.659, df = 2, $p = 0.008$) and soil surface temperature (Kruskal-Wallis chi-squared = 65.156, df = 2, $p < 0.001$) above nests differed significantly among the three habitats.

Table S4. Temperature variation within a nest at open, filtered, and fully-shaded habitats.

	Top	Middle	Bottom
Open habitat	29.25 ± 0.13°C (n = 2304)	27.97 ± 0.09°C (n = 2304)	27.58 ± 0.08°C (n = 2304)
Filtered habitat	28.55 ± 0.12°C (n = 2304)	27.57 ± 0.09°C (n = 2304)	27.66 ± 0.08°C (n = 2304)
Shaded habitat	27.41 ± 0.09°C (n = 2304)	27.39 ± 0.08°C (n = 2304)	26.97 ± 0.08°C (n = 2304)

The Kruskal-Wallis test was used to evaluate the air and surface temperatures of semi-natural experimental sites, and the temperature heterogeneity of different nest locations, data show as mean ± SE. Within a nest at each habitat, temperatures were higher at the top of nests than at the middle and bottom of nests (open, Kruskal-Wallis chi-squared = 36.307, df = 2, $p < 0.001$; filtered, Kruskal-Wallis chi-squared = 16.372, df = 2, $p < 0.001$; and fully-shaded, Kruskal-Wallis chi-squared = 12.788, df = 2, $p = 0.002$). Nest temperatures were the highest in open habitats, the lowest in fully-shaded habitats, with filtered habitat in between (Kruskal-Wallis chi-squared = 82.541, df = 2, $p < 0.001$).
