# Supplementary material for: Behavioral thermoregulation by reptile embryos promotes hatching success and synchronization
Source: Commun Biol. 2023 Aug 15;6:848. doi: 10.1038/s42003-023-05229-8 (PMC10427690; doi:10.1038/s42003-023-05229-8)
Supplement: Supplementary file 3 — Description of Additional Supplementary Files [file 42003_2023_5229_MOESM3_ESM.pdf]

## Description of Additional Supplementary Files

**File name:** Supplementary Data 1

**Description:** This data file contains 3 independent sheets representing all the data from this study (sheet of Experiment A: Effects of capsazepine on embryonic development and hatchling traits; sheet of Experiment B: Effects of behavioral thermoregulation on incubation period and hatchling traits; and sheet of Experiment B's temperature data,). We get Table S1 from the data sheet of Experiment A; Table 1, Figure 1, Figure 2 & Table S2 from the data sheet of Experiment B; Table S3 & Table S4 from the data sheet of Experiment B's temperature data)
