# Supplementary material for: Behavioral thermoregulation by reptile embryos promotes hatching success and synchronization
Source: Commun Biol. 2023 Aug 15;6:848. doi: 10.1038/s42003-023-05229-8 (PMC10427690; doi:10.1038/s42003-023-05229-8)
Supplement: Supplementary file 5 — Reporting Summary [file 42003_2023_5229_MOESM5_ESM.pdf]

nature portfolio

Corresponding author(s): Weiguo Du

Last updated by author(s): Jul 23, 2023

Reporting Summary

Nature Portfolio wishes to improve the reproducibility of the work that we publish. This form provides structure for consistency and transparency in reporting. For further information on Nature Portfolio policies, see our Editorial Policies and the Editorial Policy Checklist.

Statistics

For all statistical analyses, confirm that the following items are present in the figure legend, table legend, main text, or Methods section.

n/a	Confirmed	
☐	☒	The exact sample size (*n*) for each experimental group/condition, given as a discrete number and unit of measurement
☐	☒	A statement on whether measurements were taken from distinct samples or whether the same sample was measured repeatedly
☒	☐	The statistical test(s) used AND whether they are one- or two-sided<br>*Only common tests should be described solely by name; describe more complex techniques in the Methods section.*
☐	☒	A description of all covariates tested
☐	☒	A description of any assumptions or corrections, such as tests of normality and adjustment for multiple comparisons
☐	☒	A full description of the statistical parameters including central tendency (e.g. means) or other basic estimates (e.g. regression coefficient) AND variation (e.g. standard deviation) or associated estimates of uncertainty (e.g. confidence intervals)
☐	☒	For null hypothesis testing, the test statistic (e.g. F, t, r) with confidence intervals, effect sizes, degrees of freedom and P value noted<br>*Give P values as exact values whenever suitable.*
☒	☐	For Bayesian analysis, information on the choice of priors and Markov chain Monte Carlo settings
☒	☐	For hierarchical and complex designs, identification of the appropriate level for tests and full reporting of outcomes
☒	☐	Estimates of effect sizes (e.g. Cohen's *d*, Pearson's *r*), indicating how they were calculated

Our web collection on statistics for biologists contains articles on many of the points above.

Software and code

Policy information about availability of computer code

Data collection	The temperature data, the weight of the Chinese soft-shelled turtle eggs and the data (developmental stage of the embryos, the hatching time of the Chinese soft-shelled turtle hatchlings, the weight, the length of the carapace, and the width of the carapace) were measured by our team. No software was used to generate the data.
Data analysis	IBM SPSS Statistics Version 26.0 (IBM Corp)

For manuscripts utilizing custom algorithms or software that are central to the research but not yet described in published literature, software must be made available to editors and reviewers. We strongly encourage code deposition in a community repository (e.g. GitHub). See the Nature Portfolio guidelines for submitting code & software for further information.

Data

Policy information about availability of data

All manuscripts must include a data availability statement. This statement should provide the following information, where applicable:

- Accession codes, unique identifiers, or web links for publicly available datasets
- A description of any restrictions on data availability
- For clinical datasets or third party data, please ensure that the statement adheres to our policy

All data needed to evaluate the conclusions are present in this paper and/or the supporting information.

Human research participants

Policy information about studies involving human research participants and Sex and Gender in Research.

Reporting on sex and gender	Not involved.
Population characteristics	Not involved.
Recruitment	Not involved.
Ethics oversight	Not involved.

Note that full information on the approval of the study protocol must also be provided in the manuscript.

Field-specific reporting

Please select the one below that is the best fit for your research. If you are not sure, read the appropriate sections before making your selection.

☐ Life sciences ☐ Behavioural & social sciences ☒ Ecological, evolutionary & environmental sciences

For a reference copy of the document with all sections, see nature.com/documents/nr-reporting-summary-flat.pdf

Ecological, evolutionary & environmental sciences study design

All studies must disclose on these points even when the disclosure is negative.

Study description	Our study controls embryonic behavioral thermoregulation by using Capsazepine to cause embryos undergoing a semi-natural incubation environment, and by measuring pipping time and phenotypic indicators of hatchlings to complement the adaptive significance of behavioral thermoregulation by embryos.
Research sample	In July 2021, we obtained 15 clutches of 210 fertilized eggs produced on the same day at a private turtle farm in Tangshan, Hebei Province, China, and also measured the depth (sand substrate) of 70 natural Chinese turtle nests. These eggs eventually hatched 182 individuals (Capsazepine treatment group: n = 92; control group: n = 90), and we measured their piping time, body weight, carapace length, carapace width, and righting time (all samples were equal, Capsazepine treatment group: n = 92; control group: n = 90), respectively. 73 clutches of Chinese soft-shelled turtles produced on the same day were obtained at the same private turtle farm in May 2022 and we obtained 1066 embryos (442 eggs from 28 clutches used to determine the embryonic developmental stages; 624 eggs from 45 clutches used to investigate the effects of thermoregulation by embryo on the hatching period and hatchling characteristics in the Chinese soft-shelled turtle). Among these eggs, data of 272 embryonic stages were obtained from 442 eggs (behavioral thermoregulation group: n = 135; behavioral thermoregulation inhibited group: n = 137); 418 hatchlings were hatched from 624 eggs (behavioral thermoregulation group: n = 203; behavioral thermoregulation inhibited group: n = 215), and their pipping times and hatchling weights were measured separately (same sample size, behavioral thermoregulation group: n = 203; behavioral thermoregulation inhibited group: n = 215) and righting time (behavioral thermoregulation group: n = 202; behavioral thermoregulation inhibited group: n = 214). Eggs produced on the same day were selected to ensure that the timing and state of initiation of development was similar for all embryos. The depth of the nest was measured to simulate a natural nest setting for artificial nests. Data for all hatchling phenotype measurements were obtained from the laboratory located at the Institute of Zoology, Chinese Academy of Sciences, Beijing. All air temperature (n = 2304), soil surface temperature (n = 2304), and nest temperature (n = 20736) data were obtained from a semi-natural experimental field in Zhoushan, Zhejiang Province, China. The embryos of the Chinese soft-shelled turtle used in this study involved only the identification of embryonic stages and phenotypic measurements of hatchlings.
Sampling strategy	In our study, we ensured that the total number of eggs used for the experiment was not less than 15 for each treatment group (with or without the Capsazepine treatment applied). We were able to centrally obtain the egg weight of each fertilized egg at the beginning of the experiment (2021: n = 210; 2022: n = 1066), which can be used as a covariate to facilitate our mixed linear model analysis of hatchling weight and carapace size. We obtained data of embryo developmental stages during embryo development (six separate samplings, one egg from each treatment group in all of 28 clutches, with no less than 15 eggs sampled from each group in a separate sampling), which allowed us to compare the differences in embryo developmental stages between two groups using the Mann-Whitney U tests. After embryo hatching, we were able to obtain data on incubation periods, body mass, carapace length, carapace width, and righting time for the hatchlings in a more centralized manner, which allowed us to sample all data completely and thus to analyze hatching time and righting time using a generalized linear mixed model with a large sample size (>30 for all measures in each treatment group). Our hourly measurements of nest temperature, soil surface temperature, and air temperature under different shadow cover were also sufficient for us to compare microhabitat differences with daily fluctuations in temperature using the Kruskal-Wallis test.
Data collection	Shuo Liu measured nest depth data by digital caliper (CD-6" CSX, Mitutoyo, Japan), collected developmental stages data by embryo dissection, obtained temperature data by Thermal data-loggers (iButton, DS1921G; MAXIM Integrated Products Ltd., USA), and obtained righting time data by video camera (DCR-SR220E; Sony, Japan). Xiao-Ting Gu obtained embryo pipping time by live cameras, egg mass and hatchling weight by electronic balance, and carapace size data by digital caliper.

Timing and spatial scale	We obtained all egg weight data on two days (July 21, 2021; May 29, 2022), in order to serve as covariates for hatchling weight analysis and to obtain mean values of all egg weights. Beijing, China.<br>We collected temperature data from the field and nests during the period between June 11, 2022 to July 12, 2022. Hourly temperature loggers record real-time temperatures so that we may capture daily variation in hatchery temperatures. We also collected data on the developmental stages of 28 clutches of Chinese soft-shelled turtle embryos. Embryos from different treatment groups are sampled weekly, which allows us to confirm the speed of embryo development. Zhoushan, Zhejiang Province, China.<br>We collected the time of hatchling shell breakage and their body mass, hatching success, righting time, carapace size (length and width) and calculated embryos' incubation periods, coefficient of variation, variation range from different treatment groups during September 1, 2021 to September 6, 2021; we also collected the time of hatchling shell breakage and their body mass, hatching success, righting time and calculated embryos' incubation periods, coefficient of variation, variation range from two groups during July 14, 2022 to July 31, 2022. The time of hatchling shell breakage was monitored by different people in turn or the live cameras, and the body mass and righting time of each hatchling was measured within 24 hours after hatching. In this way, the exact time of shell breakage and more accurate phenotypic data of each hatchling can be specifically obtained. Beijing, China.
Data exclusions	In this study, all embryos that died during incubation identified by cold light were removed from the data they represented because the dead embryos could not hatch. The data represented by all hatchlings that could not complete the righting test within 10 minutes were removed in the comparison of the difference in righting time, a determination that was possible in a previous study (Ye et al. 2012, Current Biology)
Reproducibility	Our experimental design ensures sufficient clutch size (not less than 15 clutches for each treatment) and sufficient embryos (not less than 12 eggs per clutch). In the 2022 experiment, our results showed similar results and trends even under different habitats. Relevant results have been included in the supplemental material of the manuscript.
Randomization	In the comparison of the differences of all samples from the two treatment groups (with or without the application of Capsazepine) that we studied, the total sample of each group was obtained from a collection of multiple clutches of eggs, which greatly ensures randomness.
Blinding	In our study, data collection and analysis were barely done by one person.

Did the study involve field work? ☒ Yes ☐ No

Field work, collection and transport

Field conditions	From May 30, 2022 to July 13, 2022, we conducted experiments in which embryos experienced natural temperature in an unused farm land located in Zhoushan, China. During this period, the weather conditions at the experimental field were highly variable, with sunshine, cloudy skies, rainfall and fog. The average air temperature ranged from 20ºC to 28 ºC.
Location	The field experiment was done in Zhoushan, Zhejiang Province (29.99N, 122.27E). The experimental field contains herbaceous plants and some small and medium-sized shrubs. Our filed was located next to a ditch with a water depth of about 35 cm.
Access & import/export	Our filed experiment makes use of private unused farm land, and we have obtained farmers' consent and short-term land use rights before conducting the experiment.
Disturbance	We set up 73 artificial nests (average depth about 10 cm) in the field, and we leveled the filed after the incubation experiment. In addition, any garbage produced during the experiments (ie, plastic signs for nests and experimental gloves), we followed the local principles of waste separation and disposal.

Reporting for specific materials, systems and methods

We require information from authors about some types of materials, experimental systems and methods used in many studies. Here, indicate whether each material, system or method listed is relevant to your study. If you are not sure if a list item applies to your research, read the appropriate section before selecting a response.

Materials & experimental systems

n/a	Involved in the study	
☒	☐	Antibodies
☒	☐	Eukaryotic cell lines
☒	☐	Palaeontology and archaeology
☐	☒	Animals and other organisms
☒	☐	Clinical data
☒	☐	Dual use research of concern

Methods

n/a	Involved in the study	
☒	☐	ChIP-seq
☒	☐	Flow cytometry
☒	☐	MRI-based neuroimaging

Animals and other research organisms

Policy information about studies involving animals; ARRIVE guidelines recommended for reporting animal research, and Sex and Gender in Research

Laboratory animals	This study did not involve laboratory animals.
Wild animals	This study did not involve wild animals.
Reporting on sex	All our experiments do not involve any determination of sex.
Field-collected samples	This study did not involve samples collected from field.
Ethics oversight	This study was performed under approvals from the Animal Ethics Committee at the Institute of Zoology, Chinese Academy of Sciences (IOZ14001).

Note that full information on the approval of the study protocol must also be provided in the manuscript.
